# The Role of Genetic Variation in Modulating the Effects of Blended Fruits and Vegetables Versus Fruit- and Vegetable-Coated Food Products on Antioxidant Capacity, DNA Protection, and Vascular Health: A Randomized Controlled Trial

**DOI:** 10.3390/nu17122036

**Published:** 2025-06-18

**Authors:** Julia N. DeBenedictis, Na Xu, Theo M. de Kok, Simone G. van Breda

**Affiliations:** Department of Translational Genomics, Faculty of Health, Medicine & Life Sciences, GROW Institute for Oncology and Reproduction, Maastricht University, P.O. Box 616, 6200 MD Maastricht, The Netherlands

**Keywords:** fruit and vegetable mixtures, human intervention study, genetic variability, phytochemical enriched foods, chronic disease prevention, antioxidant activity

## Abstract

**Background/Objectives**: Fruits and vegetables (F&Vs) are major dietary sources of phytochemicals, crucial for preventing non-communicable diseases. However, barriers such as preparation inconvenience and a short shelf life hinder their consumption. F&V-coated foods have emerged as an alternative. This human nutrition intervention study assessed the effects of a blended F&Vs mixture versus an F&V-coated food on phytochemical absorption and chronic disease risk markers. It also explored how genetic variation influences physiological responses to these F&V products. **Methods**: In this randomized-controlled trial, participants were assigned to one of three dietary interventions: a blended F&V mixture (“F&V Blend”), a rice-based cereal product coated with this blend (“Coated Pearl”), or the same product without the F&V mixture (“Uncoated Pearl”). The four-week study included a two-week run-in and a two-week intervention phase, each followed by a test day. Measurements included DNA damage resistance (comet assay), plasma antioxidant status (Trolox capacity and superoxide levels), microvasculature health (retinal analysis), and plasma phytochemical concentrations (colorimetric analyses or HPLC). To assess group differences, a linear mixed model was used. Fifteen polymorphic genes related to phytochemical metabolism and oxidative stress were tested using TaqMan and PCR, with outcomes analyzed via ANOVA. **Results**: The F&V Blend and Coated Pearl products increased plasma carotenoid levels versus the Uncoated Pearl product. Only the F&V Blend improved retinal dilation and DNA resistance. Surprisingly, the Uncoated Pearl product enhanced antioxidant capacity, lowered superoxide levels, and improved retinal microvasculature. Genotype effects were minimal, except for HNF1A, where wildtypes in the Uncoated Pearl group showed a higher antioxidant capacity. **Conclusions**: Fresh F&Vs were more effective than coated alternatives in improving vascular health and DNA protection.

## 1. Introduction

Diet and lifestyle changes in industrialized nations have contributed to the rising burden of non-communicable diseases, including metabolic, cardiovascular diseases, and cancer. This is marked by an increasing consumption of ultra-processed foods and a decline in the intake of fruits and vegetables (F&Vs) [1]. Beyond the essential fiber and micronutrients found in F&Vs, these foods offer additional health benefits through bioactive compounds known as phytochemicals. Phytochemicals are known to have antioxidant effects and can target and regulate gene transcription, promoting enhanced resilience against oxidative stress and chronic disease development [2,3,4].

However, processed foods often take precedence over whole F&Vs in the modern diet due to their convenience, extended shelf life, reduced meal preparation time, and minimized food waste. Moreover, these processed options are often energy-dense and highly salient, encouraging repeated consumption [5]. One potential way to shift these foods from being disease-promoting to health-supporting is to enhance the nutritional value of processed foods with F&Vs [6].

Dietary interventions often yield varying responses, with some individuals experiencing more pronounced improvements compared to others [7,8]. One factor contributing to this diversity in responses is the genetic makeup of the participants. Genetic differences, specifically variations in the base pairs of certain protein-coding genes, known as genetic polymorphisms, have been extensively studied for their influence on physiological responses [9]. These responses include the body’s ability to detoxify harmful compounds, withstand oxidative stress, and absorb and metabolize phytochemicals. In a previous study, we demonstrated that an individual’s genotype for particular genes can significantly impact their response to a dietary intervention, affecting the likelihood of deriving substantial benefits from specific nutrients [10]. Therefore, in the current study, we anticipate identifying individuals with particular single nucleotide polymorphism (SNP) variants who may experience more substantial physiological improvements when consuming phytochemical-enriched foods compared to others.

We therefore performed a human nutrition intervention study aiming to explore both the effect of infusing a novel, dry food product with F&V on DNA strand breaks, oxidative stress, phytochemical absorption, and retinal microvasculature and which individuals, based on genetic background, are more responsive to this type of intervention. We address these aims by comparing three intervention groups. One group will consume a complex blend of whole F&Vs, while another group will consume a dry food product based on rice flour, coated with an equivalent amount of the same F&V Blend. A third control group will consume the dry food product without an F&V infusion. This comparative analysis will shed light on how the effects of an F&V-enriched food product compares to traditional whole F&V consumption. It will also help us discern the potential contributions of the ingredients in the dry food product. To investigate the link between genetic variability and individual responses to the intervention, we will analyze participant genotypes for SNPs related to nutrient metabolism and chronic disease risk. This approach allows us to identify individuals who may experience heightened benefits from an F&V-coated dry food product, thereby prioritizing them for targeted dietary recommendations.

Selected to reflect modifications in chronic disease risk from dietary changes, the chosen endpoints include DNA damage susceptibility, oxidative stress, microvasculature health, and the absorption of bioactive compounds. Chronic oxidative stress results from an imbalance between ROS production and antioxidant defenses and is a major contributor to non-communicable diseases, including cardiovascular disease and cancer. F&V-derived antioxidants support redox homeostasis through both direct and indirect mechanisms. For instance, vitamin C and phenolic compounds can scavenge free radicals directly, while others (such as sulforaphane or curcumin analogs) activate transcription factors like Nrf2, which upregulate endogenous antioxidant enzymes (e.g., glutathione peroxidase, superoxide dismutase). Moreover, dietary carotenoids have been shown to localize within cell membranes, where they prevent lipid peroxidation, thus preserving membrane integrity and cellular function [11]. Given that oxidative stress plays a pivotal role in driving the development of chronic diseases, we assessed the Trolox-equivalent antioxidant capacity (TEAC) of plasma and measured superoxide levels in whole blood [12]. The evaluation of DNA damage, measured by the comet assay on participant lymphocytes ex vivo, provides insights into susceptibility to oxidative stress-induced DNA strand breaks—a key risk factor in the development of non-communicable diseases such as cancer [13]. Many F&V-derived compounds, particularly polyphenols and carotenoids (e.g., quercetin, lutein, β-carotene), exhibit potent antioxidant properties that help prevent DNA strand breaks by neutralizing reactive oxygen species (ROS). Additionally, some phytochemicals have been shown to modulate the expression and activity of DNA repair enzymes, such as OGG1 and XRCC1, thereby enhancing the cell’s capacity to repair oxidative lesions. Certain flavonoids may also protect DNA indirectly by inhibiting pro-oxidant enzymes (e.g., NADPH oxidase) or reducing chronic inflammation, a known driver of mutagenesis [14,15]. The assessment of retinal microvasculature arteriolar and venular diameter serves as an indicator of microvasculature health, offering valuable information on the risk of cardiovascular diseases, diabetes, and hypertension [16]. Polyphenols such as flavanols and anthocyanins improve endothelial function by increasing nitric oxide (NO) availability, reducing inflammation, and enhancing capillary perfusion [17,18]. Bioactive compound absorption was measured through an analysis of plasma polyphenol, carotenoid, and urinary vitamin C levels. These levels are negatively correlated with the risk of developing neurodegenerative diseases, cardiovascular diseases, and cancer [19,20,21].

Lastly, the study delves into the participants’ genetic landscape by assessing 15 polymorphisms (13 SNPs and 2 deletion polymorphisms) linked to chronic disease risk and phytochemical metabolism [22]. This is a relevant consideration, as genetic polymorphisms in metabolizing enzyme genes, for example, can affect the enzyme’s efficiency, leading to ‘slow’ or ‘fast’ metabolizers of particular nutrients or classes of compounds [23]. This distinction has important consequences for phytochemical metabolism and the body’s ability to resist oxidative stress. ‘Slow metabolizers’ may experience reduced biological effects from phytochemicals that require activation and less resilience to oxidative stressors. However, slower metabolizing enzymes may also lead to sustained physiological impacts from other absorbed phytochemicals or a harmful build-up of genotoxic compounds in the case of slow detoxification enzymes [24]. This inter-individual genetic variation could influence study outcomes and explain diverse responses to the same dietary nutrients.

We therefore hypothesize that the F&V-coated product produced using methods that aim to preserve bioactive compound concentrations will perform similarly to the fresh F&V Blend in improving these markers of chronic disease risk, whereas the uncoated processed product will not induce a change in these levels.

## 2. Materials and Methods

### 2.1. Study Design

**Figure 1:**
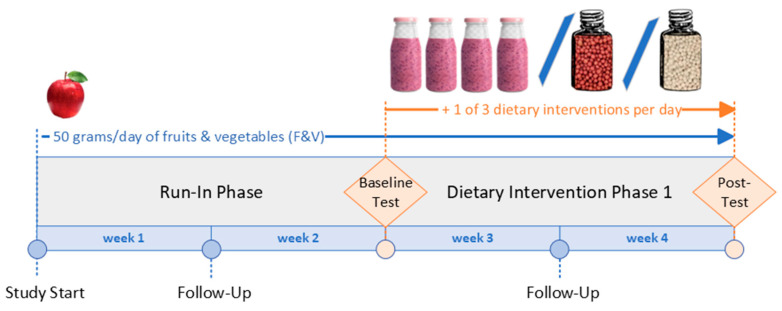
Dietary investigation timeline. A randomized-controlled trial assessing the effects of three dietary interventions: a blended fruits and vegetables mixture (F&V Blend), a rice-based cereal product coated with the F&V Blend (Coated Pearl), and an uncoated version of the rice-based product (Uncoated Pearl). The study consisted of a two-week run-in phase where participants consumed 50 g of F&Vs daily, followed by a two-week intervention phase where participants continued their baseline diet and were randomized to consume 400 g of the F&V Blend, 133 g of the Coated Pearl, or 100 g of the Uncoated Pearl daily. Participants’ compliance was monitored through digital food diaries, weekly check-ins, and periodic collection of study materials. Blood, urine, retinal images, and anthropometric measurements were taken at baseline and post-intervention to assess health outcomes.

This investigation utilizes a randomized-controlled trial design where participants were randomized to one of three dietary interventions (DIs). The interventions include a complex, blended mixture of F&Vs (“F&V Blend”), a dry cereal-like food product based on rice flour coated with this blended mixture (“Coated Pearl”), and the same dry food product without the F&V Blend (“Uncoated Pearl”). The study was four weeks long, with a two-week run-in phase and a two-week intervention phase, each followed by a test day where measurements were taken (Figure 1). During the run-in phase, participants only consumed 50 g of F&Vs per day. During the intervention phase, they continued this low-F&V diet in addition to consuming their randomized intervention each day for two weeks.

Participants were randomized to the F&V Blend group and consumed 400 g per day of this F&V mixture. The Coated Pearl contained the equivalent of 400 g of the F&V Blend coated onto 100 g of a dry food product, resulting in a total of 133 g of final product. Therefore, participants randomized to the Coated Pearl group consumed 133 g of this intervention per day. Those randomized to the Uncoated Pearls, consumed 100 g per day. In this manner, the groups that had the F&V Blend and the Coated Pearl both consumed 400 g of F&V per day, and those that had the Coated Pearl and the Uncoated Pearl both consumed 100 g of the basic pearl per day.

Participants maintained a digital food diary that was routinely reviewed by a Registered Dietitian Nutritionist. Intake of phytochemical-rich foods was controlled throughout the study, with a restriction of no more than two cups of coffee or tea per day. The consumption of dietary supplements, real fruit juice, red wine, and green tea was not allowed. For participants who consumed alcohol, high-risk drinking was restricted. Female participants were limited to a maximum of two alcoholic beverages per day, up to twice per week, while male participants were allowed up to three drinks per day, no more than three times per week.

Participants reported to the testing site in the morning on both the baseline and post-intervention days to provide fasting blood samples and submit urine samples collected over the prior 24 h. Additionally, a photograph of the fundus of their right eye was taken, and their height and weight were measured. Outside of test days, participants attended weekly follow-up visits to meet with the study coordinator for check-ins and to exchange study materials. During visits when interventions were provided, participants received a one-week supply. At the following visit, they returned their used bottles, which the study coordinator reviewed to assess compliance.

### 2.2. Dietary Interventions

**Figure 2:**
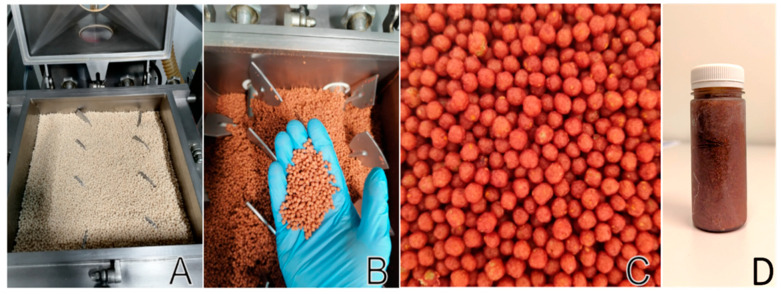
(**A**) Uncoated Pearls; (**B**) Pearls coated with complex F&V Blend; (**C**) close-up view of Coated Pearls; (**D**) F&V Blend (100 g). The F&V Blend was created by blending apples (100 g), green tea (0.5 g in 25 mL water), blueberries (25 g), blue grape (25 g), blackberries (25 g), raspberries (25 g), tomato (33 g), carrots (33 g), red bell pepper (33 g), broccoli (33 g), cauliflower (33 g), Brussels sprouts (33 g) (a total of 400 g), followed by bottling and Pascalization using high-pressure, low-heat technology. The Uncoated Pearls were dry, cereal-like spheres composed of 95% rice flour and 5% oat flour. The Coated Pearls were identical in composition to the Uncoated Pearls but coated with 400 g of the Pascalized F&V Blend. Preparation described in detail in DeBenedictis et al. (2023) [25].

The interventions were prepared as described in DeBenedictis et al. (2023) [25]. Briefly, all interventions were prepared at MiFood B.V. (Venlo, The Netherlands). The F&V Blend was created by blending the F&Vs listed in Table 1. The raw ingredients used for this study were commercially sourced from Venlo, The Netherlands, to represent the produce accessible in the local market. The F&V Blend was then bottled and pascalized (Pascal Processing, Helmond, The Netherlands) using a high-pressure, low-heat sterilization process (Avure High Pressure Processing machine, JBT/Avure Technologies, Middletown, OH, USA). This involved applying 6000 bars of pressure for 3 min at temperatures below 25 °C. The Uncoated Pearl was the dry-food product, called Pearls, made of 95% rice and 5% oat flour formed into homogenous cereal-like spheres (Figure 2) (Extruded Cereal Products B.V., Helmond, The Netherlands). As the rice flour was not milled as whole grains and therefore did not contain bran or germ, it was not expected to contain notable amounts of phytochemicals. However, a small percentage of the oat flour was expected to contain some bioactive ingredients. The Coated Pearl is the same dry product, but with the Pascalized mixture (F&V Blend) vaporized and coated into it. The coating process was performed using a Pegasus^®^ Vacuum Coater (Dinnissen B.V., Sevenum, The Netherlands) under vacuum conditions (90 mbar). The F&V Blend was introduced into the kettle in vapor form. The pressure reduction caused the blend’s solid particles to infuse into the Pearl matrix while the moisture evaporated. This method allowed for 400 g of fresh fruits and vegetables to be infused into every 100 g of Pearls. The coated Pearls were produced using low-temperature extrusion technology, which helps preserve both the quantity and quality of phytochemicals.

The F&V Blend was split into 100 g bottles and participants were given four bottles per day to encourage them to consume the F&V Blend throughout the day rather than all at once. All dietary interventions were prepared in a single batch and kept frozen at −18 °C [25].

Each week, the study coordinator retrieved 28 bottles of the F&V Blend from frozen storage and thawed them at 4 °C over a 48 h period before distribution. During the intervention phases, participants were instructed to store the bottles in a cold environment for as long as possible. The Pearl interventions, Uncoated and Coated, were kept at room temperature and in the dark. One bottle per day of 100 g of Uncoated Pearls or 133 g of Coated Pearls were given to participants weekly, and they were encouraged to consume the Pearls throughout the day, similar to the F&V Blend. They also returned the empty bottles at the next follow-up appointment.

**Figure 3:**
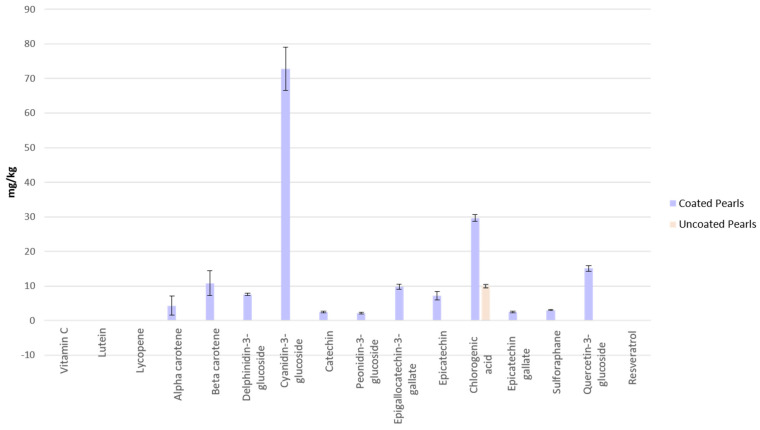
Phytochemical content in Coated and Uncoated Pearls. Data is presented as means ± SEM. Detailed descriptions of measurements of phytochemicals are provided in DeBenedictis et al. (2023) [25].

**Figure 4:**
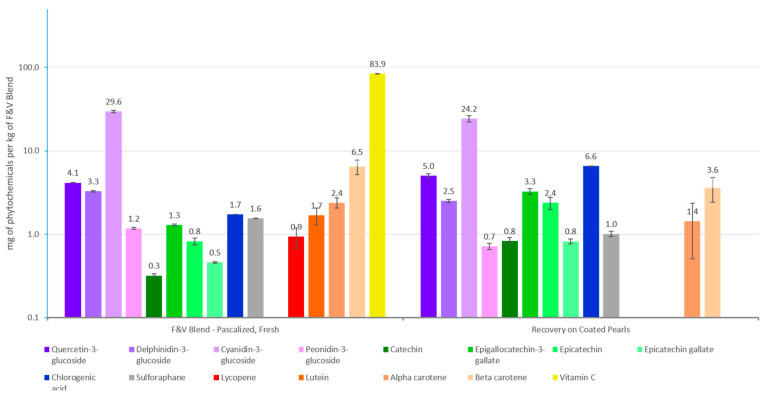
Comparison of phytochemical concentrations in the F&V Blend versus Coated Pearls. Data is presented as means ± SEM. Detailed descriptions of measurements of phytochemicals are provided in DeBenedictis et al. (2023) [25].

The concentrations of a select number of phytochemicals were measured in the Pearls prior to the experiment (Figure 3) [25]. The Uncoated Pearls only contained chlorogenic acid, compared to the Coated Pearls, which contain all of the phytochemicals included in the F&V Blend (Figure 4), except for vitamin C, lutein, and lycopene.

### 2.3. Participants

Participants were recruited using online platforms and printed flyers distributed in Maastricht, The Netherlands. The study received ethical approval from the Medical Ethics Review Committee at Maastricht University Medical Centre+ (MUMC+), under the registration number NL66118.068.18. It was registered with the International Trial Registry Platform (ICTRP) under ID NL7358 and conducted in accordance with the Declaration of Helsinki.

Prospective participants were given a minimum of one week to consider their involvement before signing informed consent forms. Written consent was obtained from all individuals, followed by the completion of a medical history questionnaire prior to the study’s initiation. At the start of the study, participants were trained on how to record their daily food intake using a food diary, which was reviewed weekly to ensure compliance and accuracy.

Eligible participants were healthy individuals between the ages of 18 and 60, with a body mass index (BMI) between 18.5 and 27 kg/m^2^. Individuals were excluded if they had a history of alcohol abuse within six months before the study, were current smokers or had quit smoking within the previous three months, or had any diagnosed or symptomatic diseases involving the gastrointestinal tract, kidneys, liver, heart, lungs, or conditions affecting the endocrine or metabolic systems. Additional exclusion criteria included HIV, hepatitis, anemia, pregnancy, recent use of antibiotics (within the past three months), current use of medications other than contraceptives, food allergies relevant to the dietary intervention, adherence to vegetarian or vegan diets, engaging in high levels of physical activity (more than eight hours per week of vigorous exercise), or participation in other intervention studies at the time.

### 2.4. Test Days

On each test day, the study coordinator reviewed the participant’s food diary and collected their 24 h urine container(s). The urine samples were weighed, thoroughly mixed, divided into aliquots using freezer-safe Eppendorf tubes, and stored at −20 °C for later analysis of 8-OHdG, 8-isoprostane, and vitamin C. Afterward, the participant’s height and weight were recorded, with their baseline weight used to determine BMI (kg/m^2^).

A fasting blood sample was then drawn using EDTA-coated Venoject II tubes (Terumo-Europe, Leuven, Belgium). Portions of the blood were immediately processed for RNA and superoxide preservation, while the remaining sample was kept on ice for either storage at −80 °C or delivery to the Maastricht University Medical Center+ clinical diagnostic laboratory for blood composition analysis.

Finally, a trained staff member captured an image of the participant’s right eye using a Canon CR-2 non-mydriatic retinal camera (Serial No. 103138), which was archived for later evaluation of retinal microvasculature. All procedures on test days were carried out in a fixed sequence to maintain consistency across visits.

### 2.5. DNA Strand Breaks

To evaluate the protective effects of the various interventions against DNA damage, the alkaline comet assay was employed in accordance with the most recent guidelines [26,27]. This assay was conducted on freshly isolated lymphocytes collected on the same day as the test-day blood draw. Lymphocyte samples were adjusted to a concentration of 1 × 10^6^ cells/mL and exposed to 25 µM hydrogen peroxide (H_2_O_2_; Merck, Darmstadt, Germany). Each treated sample was incubated alongside a non-exposed control for one hour at 37 °C. Both sets of samples were then embedded in triplicate onto microscope slides using 0.65% low melting point agarose (Sigma-Aldrich, Steinheim, Germany). Slides were lysed overnight at 4 °C in a high-pH (pH 10) lysis buffer containing 2.5 M NaCl, 100 mM EDTA, 10 mM Tris, and 250 mM NaOH (all from Sigma-Aldrich, Steinheim, Germany). Just before use, the buffer was supplemented with 10% DMSO and 1% Triton X-100. Following lysis, the DNA was allowed to unwind in an electrophoresis buffer consisting of Milli-Q water, 300 mM NaOH, and 1 mM EDTA for 40 min, after which electrophoresis was carried out at 1 V/cm for 20 min. Slides were then rinsed with 1×PBS and ethanol, air-dried, and stored in light-protective holders at 4 °C. For analysis, slides were stained with 10 µg/mL ethidium bromide (Cleaver Scientific, Rugby, UK), covered with coverslips, and imaged after one hour using a BIO-TEK Cytation 3 imaging reader with Gen5 software. The resulting images were stored and analyzed using the Comet Assay IV software (Instem). For each participant, 50 comets per condition were evaluated, and the average percentage of Tail DNA and Tail Moment from triplicate samples was recorded. All assay and scoring procedures were conducted by the same researcher to minimize variability between experiments and scorers.

### 2.6. Oxidative Stress Markers

#### 2.6.1. Antioxidant Capacity

The Trolox equivalent antioxidant capacity (TEAC) assay was used to determine the total antioxidant capacity in plasma of all volunteers, according to the procedures described by Fischer et al. (2005) [28]. Plasma samples collected from participants were retrieved from −80 °C storage and thawed for analysis. A phosphate buffer was prepared weekly by dissolving sodium phosphate monobasic (Merck, Darmstadt, Germany) in Milli-Q water and adjusting the pH to 7.4 using 1 M sodium hydroxide (Merck, Darmstadt, Germany). To create the radical solution, 2,2′-azinobis (3-ethylbenzothiazoline-6-sulfonic acid) diammonium salt (ABTS; Sigma-Aldrich, Steinheim, Germany; CAT: A1888) was dissolved in the prepared phosphate buffer. Separately, 2,2′-azobis-(2-amidinopropane) dihydrochloride (ABAP; Polysciences, Inc., Warrington, PA, USA; CAT: 08963) was also dissolved in phosphate buffer. These two solutions were mixed in a flask, and an additional 50 mL of phosphate buffer was added to achieve a total volume of 70 mL. The mixture was then incubated in a 70 °C water bath for 10 min. Absorbance measurements were taken at 734 nm using an iMark microplate reader (Bio-Rad, Hercules, CA, USA) at the 8, 9, and 10 min marks to monitor the formation of the radical solution. The target absorbance for the radical solution was 0.7 ± 0.02. Once this level was reached, the solution was placed on ice for temporary storage. Prior to use in the experiment, the solution was brought back to 37 °C using a water bath. Both the radical solution and Trolox standard dilutions were freshly prepared each day.

Trolox [(±)-6-hydroxy-2,5,7,8-tetramethyl-chroman-2-carboxylic acid; Sigma-Aldrich, Steinheim, Germany; CAT: 238813] was dissolved in 39.95 mL of phosphate buffer to prepare a 1 mM stock solution. This stock was then serially diluted with phosphate buffer to generate seven Trolox standard concentrations. Phosphate buffer alone served as a blank control, and its absorbance values were subtracted from all sample readings.

To measure antioxidant activity, 280 µL of the pre-warmed (37 °C) radical solution was combined with 15 µL of participant plasma (diluted 1:2 with phosphate buffer). The mixture was dispensed into a 96-well plate using a multichannel pipette. The absorbance at 734 nm was recorded immediately (t = 0), then again after a 5 min incubation at 37 °C (t = 5). Each sample was run in triplicate. The change in absorbance between t = 5 and t = 0 for the blank was subtracted from the corresponding change in Trolox readings. A calibration curve was then generated using the change in absorbance across the Trolox standards (average R^2^ = 0.99), and this regression was used to calculate the Trolox equivalent antioxidant capacity (TEAC) of each plasma sample.

#### 2.6.2. Superoxide Scavenging

Immediately following blood collection on test days, 250 µL of whole blood was transferred from EDTA tubes into freezer-safe Eppendorf tubes and combined with 250 µL of CMH (1-hydroxy-3-methoxycarbonyl-2,2,5,5-tetramethylpyrrolidine), a spin trap specific for superoxide detection. This procedure was performed in duplicate using CMH obtained from Noxygen Science Transfer & Diagnostic GmBH (Elzach, Germany). The spin trap reagent had been pre-prepared in large batches, aliquoted, and stored at −20 °C, and then thawed shortly before use as described previously [29]. The blood–CMH mixtures were rapidly frozen using liquid nitrogen and stored at −80 °C until they were ready for analysis. Superoxide levels were determined using electron spin resonance (ESR) spectroscopy. Prior to analysis, frozen samples were thawed on ice for 50 min. From each sample, 100 µL was drawn into glass capillary tubes (Brand, Wertheim, Germany). ESR measurements were carried out at 21 °C with an EMX X-band spectrometer (Bruker, Billerica, MA, USA), using Bruker Win EPR software (version 2.11), following previously established methods [3]. Signal quantification was performed by double integration of the spectral peaks, as outlined in previous studies [29].

### 2.7. Retinal Microvasculature

In order to measure structural changes in microvasculature as an indicator for cardiovascular disease risk, the fundus of the right eye of each participant was photographed using a Canon CR-2 non-mydriatic retina camera (VITO, Mol, Belgium). To maintain consistency in analyses, all fundus images from a given participant’s test days were evaluated together using MONA-REVA software (version 3.0.0, VITO, Mol, Belgium). The key measurements obtained included the central retinal artery equivalent (CRAE), central retinal vein equivalent (CRVE), and the arteriolar-to-venular ratio (AVR). These metrics represent the average diameters of the retinal arterioles and venules, as well as their ratio, which reflects the relative width of the central retinal arteries to veins. CRAE and CRVE metrics were obtained by analyzing fundus images using a standardized annular region (1.0–3.0 times the optic disk radius). Retinal vessels were automatically segmented using a multiscale line filtering algorithm, followed by post-processing (e.g., thresholding, blob extraction). Diameters of vessels passing through the zone 0.5–1 disk diameter from the optic disk were calculated automatically, then verified and corrected by a trained researcher using the MONA REVA toolbox [30]. The software identified and measured the calibers of the six largest arterioles and venules, from which the arteriolar-to-venular ratio (AVR) was computed. To minimize interrater variability, a single researcher conducted or confirmed all vessel diameter analyses.

### 2.8. Phytochemical Absorption

#### 2.8.1. Total Polyphenols

The plasma total phenolic content was determined using the Folin–Ciocalteu method [31]. Plasma samples stored at −80 °C were thawed on ice before processing. For sample preparation, plasma was combined with 0.75 mol/L metaphosphoric acid (MPA) (Sigma-Aldrich, Steinheim, Germany), vortexed for 3 min, and then mixed with 1 mol/L hydrochloric acid (VWR). After another 2 min vortex, the mixture was incubated at 37 °C for 30 min using an Eppendorf ThermoMixer. Next, 2 mol/L sodium hydroxide was added, vortexed for 2 min, and followed by a second 30 min incubation at 37 °C. Subsequently, 0.75 mol/L MPA was added again and vortexed for 2 min. Samples were centrifuged at 20,000× *g* for 10 min, and the resulting supernatant was collected. To extract the remaining phenolics from the pellet, a 1:1 acetone–water solution (Sigma, Steinheim, Germany) was added, and the mixture was centrifuged at 2700× *g* for 10 min. This second supernatant was combined with the first. To assess total phenolics, the combined extract was mixed with Folin–Ciocalteu reagent (FCR; diluted 1:10 with distilled water; Sigma-Aldrich, Steinheim, Germany) and incubated for 5 min. Sodium carbonate (Merck, Darmstadt, Germany) was then added, and the mixture was transferred to a microplate. After a 90 min incubation at room temperature, the absorbance was read at 725 nm using a CLARIOstar Plus microplate reader (BMG LABTECH, Ortenberg, Germany). Gallic acid (Merck, Darmstadt, Germany) dissolved in 50% methanol (Honeywell, Fisher Scientific, Landsmeer, The Netherlands) was used as the standard at concentrations of 100, 50, 25, 12.5, and 6.25 μg/mL. A standard calibration curve was generated (average R^2^ = 0.99), and phenolic concentrations were expressed as milligrams of gallic acid equivalents per gram (mg GAE/g). Each sample was analyzed in triplicate.

#### 2.8.2. Carotenoids

Carotenoid levels were assessed using plasma samples previously stored at −80 °C and thawed prior to analysis. Quantification followed the protocol provided by the ClinRep HPLC Complete Kit (RECIPE Chemicals + Instruments GmbH, Munich, Germany), in line with previously published methods [32]. The analysis was carried out using a 2.5 μm C18 column (RECIPE Chemicals + Instruments GmbH), with the column temperature maintained at 60 °C. Each sample had an injection volume of 100 µL. Detection was performed at a wavelength of 450 nm with a flow rate of 0.6 mL/min. The mobile phases consisted of 97.5% methanol and 2.5% water for phase A, and isopropanol for phase B. The total run time for each sample was 20 min.

#### 2.8.3. Vitamin C

The vitamin C levels in the participants’ urine were determined using a redox titration method [33], with 2,6-dichlorophenolindophenol (DCPIP) serving as the colorimetric reagent. To prepare the DCPIP solution, DCPIP (Merck, Darmstadt, Germany) was mixed with 1.46 mM sodium acetate (Sigma-Aldrich, Steinheim, Germany) and Milli-Q water in an Erlenmeyer flask. A sodium citrate buffer was prepared by combining 62.64 mM sodium acetate with 240 mL of Milli-Q water and adjusting the pH to 3.5 using 20% phosphoric acid (VWR Chemicals BDH, VWR International B.V., Amsterdam, The Netherlands).

A vitamin C stock solution was created by dissolving 0.09 mM ascorbic acid (Sigma-Aldrich, Steinheim, Germany) in 100 mL of Milli-Q water to yield a concentration of 1.6 mg/mL. A serial dilution of this solution, including a blank, was used to generate the standard curve. For the standards, 85 µL of sodium citrate buffer and 170 µL of DCPIP were combined with 85 µL of each dilution in a 96-well plate.

Urine samples, previously stored at −80 °C, were thawed and gently vortexed. For each participant sample, 300 µL of urine was mixed with 555 µL of DCPIP, and 340 µL of this mixture was added to each well in triplicate. The microplate was covered with foil to protect it from light and placed in a CLARIOstar multi-plate reader for absorbance measurement at 520 nm, recorded over 1 min and 30 s at room temperature.

Absorbance values were adjusted using the blank control, and a standard curve (average R^2^ = 0.99) was constructed to calculate vitamin C concentrations from the triplicate average absorbance of each sample. Final vitamin C values were normalized to creatinine concentrations, which were measured by the MUMC+ clinical diagnostic laboratory. Since vitamin C degrades over time in frozen storage [25], the duration of storage (in months) was included as a covariate in the statistical analysis for this variable.

### 2.9. Selection of Gene Polymorphisms

Genetic polymorphisms were selected based on their contribution to the results of similar dietary intervention studies [7,34,35,36,37,38,39,40,41,42,43,44,45,46]. We reviewed human nutrition intervention studies that assessed outcomes related to chronic disease development, such as DNA damage and oxidative stress, or that evaluated the absorption or metabolism of phytochemicals present in the intervention products in the current study. These studies were also required to include an examination of SNPs or genetic variants in the study population. Genes were selected if they significantly influenced the participants’ outcome response and if the polymorphism’s frequency was greater than 20% in order to allow for sufficient detection in our study population [47,48]. A total of 15 polymorphisms were selected for investigation in this study population (Table 2). This included GSTM1*0, NQO1*2, CAT1*1, GSTT1*0, XRCC1*4, ZBED3, Glu298Asp, COMT, SLC23A1, MTHFR, HNF1A, GSTP1, TCF7L2, BCMO1, and APOC1.

### 2.10. Genotyping Participants

#### 2.10.1. DNA Isolation

DNA extraction was carried out using the Quick-DNA Miniprep Kit (Zymo Research, BaseClear B.V., Leiden, The Netherlands), with slight protocol modifications. Lymphocytes previously stored at −80 °C were thawed on ice. A total of 500 µL of Genomic Lysis Buffer was added to the lymphocyte pellet and gently pipetted up and down three times to mix. The sample was then incubated at room temperature for 10 min. Following incubation, the lysate was transferred to a Zymo-Spin™ IICR Column placed in a Collection Tube and centrifuged at 10,000× *g* for 1 min. The used collection tube was discarded and replaced with a fresh one. Next, 200 µL of DNA Pre-Wash Buffer was added to the column and spun at 10,000× *g* for 1 min. The flow-through was discarded, and 500 µL of g-DNA Wash Buffer was applied and centrifuged under the same conditions. The spin column was then placed into a clean 1.5 mL Eppendorf tube, the cap was opened, and the column was left to air dry for 2 min. To elute the DNA, 30 µL of Milli-Q water pre-warmed to 50 °C was added directly onto the column membrane, followed by a 5 min incubation at room temperature. DNA was then eluted by centrifugation at maximum speed for 30 s. The concentration and purity of the extracted DNA were assessed using 260/280 absorbance ratios measured on a NanoDrop Spectrophotometer (Thermo Scientific, Breda, The Netherlands). The final DNA samples were stored at −20 °C.

#### 2.10.2. Genotyping

The participants were genotyped for deletion polymorphisms (GSTT1*0 and GSTM1*0) by multiplex PCR. Single nucleotide polymorphisms (NQO1*2, CAT1*1, XRCC1*4, ZBED3, Glu298Asp, COMT, SLC23A1, MTHFR, HNF1A, GSTP1, TCF7L2, BCMO1, APOC1) were identified using the TaqMan assay (Thermo Fisher Scientific, Breda, The Netherlands).

#### 2.10.3. Multiplex PCR Assay

GSTM1 and GSTT1 polymorphisms were analyzed using multiplex PCR, with the β-globin gene serving as an internal control. Primer sequences and the corresponding amplified fragment sizes are provided in Table 3.

Primers (Eurogentec S.A., Seraing, Belgium), DNA templates, and AmpliTaq Gold^®^ 360 Master Mix (Thermo Fisher Scientific, Breda, The Netherlands) were thawed on ice prior to use. For a batch of 50 reactions, 5 µL of the specified primers (totaling 30 µL) were diluted in 70 µL of nuclease-free Milli-Q water. This was followed by the addition of 300 µL of AmpliTaq Gold Master Mix, and the mixture was gently combined. Each PCR reaction was prepared by adding 4 µL of DNA template to 8 µL of the prepared PCR mixture in 0.2 mL PCR tubes, with mixing carried out by pipetting. Tubes were sealed and loaded into a thermocycler (Westerburg) for amplification. The PCR conditions included an initial denaturation at 95 °C for 3 min, followed by 40 cycles of 95 °C for 60 s, 56 °C for 60 s, and 72 °C for 60 s, with a final extension at 72 °C for 10 min. Amplified DNA products were analyzed via electrophoresis on a 2% agarose gel. A 300 mL batch of 2% agarose gel was prepared and kept at 60 °C. From this, 20 mL was transferred to a 50 mL beaker, and 2 µL of SYBR Safe DNA gel stain (Thermo Fisher Scientific, Breda, The Netherlands) was added. The solution was gently mixed and poured into a casting tray with the comb in place. Once solidified, the gel was placed in an electrophoresis chamber filled with UltraPure™ TAE buffer (Thermo Fisher Scientific) until the gel was fully submerged. PCR products were combined with loading dye (Thermo Fisher Scientific) and carefully pipetted into the wells. Electrophoresis was conducted using a PowerPac™ Basic Power Supply (Bio-Rad) set to 140 V for 20 min. DNA bands were visualized under UV light using a D-DiGit^®^ Gel Scanner (LI-COR Biotechnology, Homburg, Germany). Band sizes were compared to a DNA ladder (Thermo Fisher Scientific) to determine the presence or absence (null genotype) of the GSTT1 and GSTM1 genes.

#### 2.10.4. Taqman Assay

The TaqMan SNP genotyping assay was executed in accordance with the established protocols provided by the manual for the TaqMan Genotyping Master Mix (Thermo Fisher Scientific, Breda, The Netherlands). The procedure was initiated by preparing a reaction mixture that included the 5 μL of 2× TaqMan Genotyping Mastermix, 0.5 μL of predesigned SNP probes (Thermo Fisher Scientific, Breda, The Netherlands) and 4.5 μL of DNA. This 10 μL mixture was then allocated to each well of a standard 96-well qPCR plate (Bio-Rad). Upon completion of the sample preparation, the 96-well plate was securely sealed using a Transparent Microplate Seal (Greiner Bio-One, Alphen aan den Rijn, The Netherlands) to prevent any contamination or evaporation during the amplification process. Following preparation, the plate was securely sealed and spun at 2000 rpm for 2 min. It was then transferred to the CFX Connect Real-Time PCR Detection System (Bio-Rad) for analysis. Channel 1 was configured to detect the FAM dye, and Channel 2 was set to monitor the VIC dye, allowing both DNA targets to be measured simultaneously in each well.

### 2.11. Sample Size Calculation, Randomization and Statistical Analysis

Details regarding sample size determination and participant randomization are available in DeBenedictis et al. (2024) [49]. Statistical analysis was conducted using linear mixed models in SPSS (IBM SPSS Statistics version 26). Each model included “Age” and “BMI” as covariates and “Sex” as a fixed factor. Least Significant Difference (LSD) corrections were applied during analysis, and *p*-values were subsequently adjusted using the False Discovery Rate (FDR) method. To evaluate the influence of genetic variation, a Univariate Analysis of Variance (ANOVA) with Tukey post hoc comparisons was conducted in SPSS. This analysis assessed the effect of gene alleles on DNA damage indicators (% tail DNA and tail moment), retinal vascular measurements (AVR), and antioxidant capacity (TEAC) across the different dietary interventions. Changes in outcomes were calculated as the difference between post-intervention and baseline values. Where applicable, both uncorrected *p*-values and FDR-adjusted q-values are reported for statistically significant results (*p* < 0.05, q < 0.05). Significant fixed effects in the models were further examined—for instance, through correlation analysis when continuous covariates were involved. Post hoc comparisons for significant effects used either independent *t*-tests (for parametric data with equal variances) or Mann–Whitney U tests (for non-parametric data with equal variances).

## 3. Results

### 3.1. Participants

**Figure 5:**
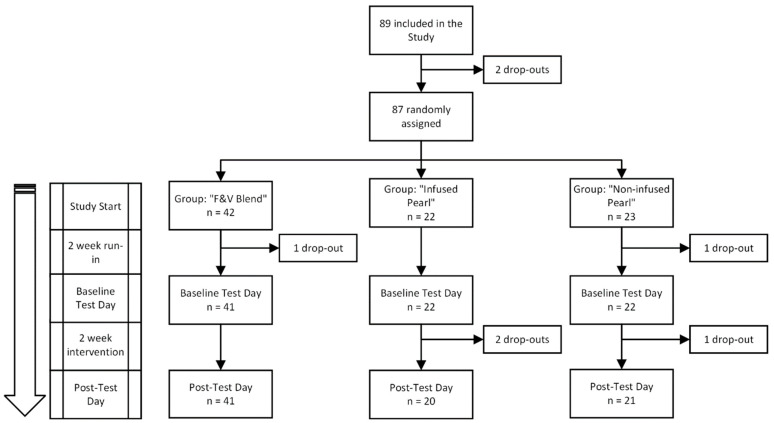
Inclusion flow chart. A total of 89 participants were enrolled in the study. After 2 dropouts, 87 participants were randomly assigned to one of three intervention groups: “F&V Blend” (n = 42), “Infused Pearl” (n = 22), and “Non-infused Pearl” (n = 23). Following a 2-week run-in period, participants underwent baseline testing. During the 2-week intervention, additional dropouts occurred: 1 from the “F&V Blend” group, 2 from the “Infused Pearl” group, and 1 from the “Non-infused Pearl” group. Final post-test data were collected from 41, 20, and 21 participants in each respective group.

The participant inclusion and dropouts in this study are shown in Figure 5. In total, 89 participants were included in the study and 82 participants completed the study between March 2019 and May 2022. The trial ended due to achieving sufficient power for each intervention group. Most dropouts were related to illness or being unable to adhere to study guidelines.

The study sample consisted of 78% female participants, with a mean age of 29 ± 11 years and an average BMI of 22.8 ± 2.0. These factors by intervention group are described in Table 4. Independent *t*-tests for age, sex, and BMI determined that no group was significantly different from another by these factors.

Food diary data was collected, and post-test measurements were compared with baseline to assess how the inclusion of the dietary interventions affected nutrient intake. There was a significant increase in sugars (average of 16 g) and fiber (average of 10 g) for those who consumed the F&V Blend. The Pearl interventions led to a slight increase in calories, with an average increase of 190 kcal for the Uncoated Pearl group and 214 kcal for the Coated Pearl group. These nutrient increases during the intervention phases were smaller than those provided by the dietary interventions themselves.

### 3.2. Phytochemical Absorption

#### 3.2.1. Total Polyphenols

There were no significant differences between the baseline and post-intervention measurements of total polyphenols in the plasma of the participants in all three groups, which does not follow expectations (Table 4). However, although not statistically different (likely due to the low power), the Uncoated Blend group’s concentration is lower than the rest, which is expected. Our experimental procedure of deproteinization removed albumin, and with it, albumin-bound polyphenols that contribute to total polyphenol concentrations [50]. Furthermore, the Folin–Ciocalteu reagent can also react with other substances co-extracted from plasma during the deproteinization process, which could have an impact on the concentrations measured.

#### 3.2.2. Carotenoids

**Figure 6:**
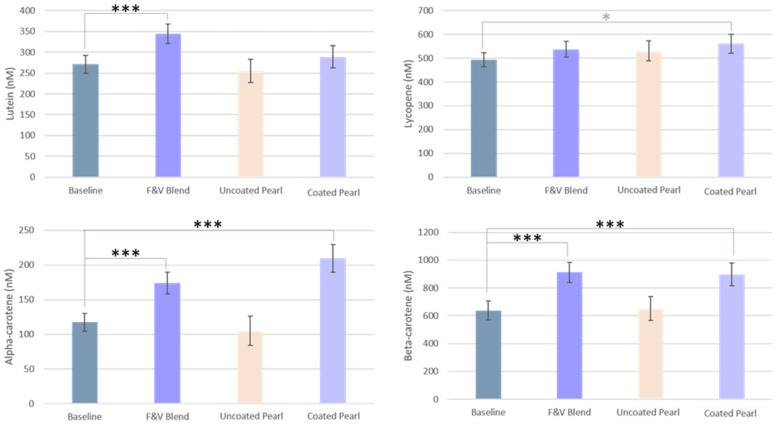
Carotenoid levels at baseline and after each intervention. Data is presented as means ± SEM. *** = *p* < 0.001; significant after FDR correction; * = *p* < 0.05, significant with LSD correction.

The plasma carotenoid concentrations in participants who consumed the F&V Blend and the Coated Pearl showed significant increases compared to baseline, whereas the Uncoated Pearl group did not differ from baseline. The lutein concentrations in the F&V Group were significantly greater than baseline. Lycopene concentrations were only significantly greater in the Coated Pearl compared to baseline before FDR correction. The measurements for alpha-carotene and beta-carotene were similar to each other. For both compounds, the F&V Blend and Coated Pearl groups had significantly increased plasma concentrations compared to baseline. (Figure 6). The inclusion of this step may have removed observable differences in plasma carotenoids concentrations between groups.

Females who consumed the Coated Pearls showed a significantly greater increase in lycopene levels (+65.1 nM + 19.7 nM) compared with males (−31.1 nM + 30.7 nM) (*p* < 0.05).

#### 3.2.3. Vitamin C

Excreted vitamin C levels measured in participant urine did not differ between the participants’ baseline and post-intervention measurements (Table 4). The Pearl groups’ values were notably lower than baseline and the F&V Blend, which was expected as these interventions were not good sources of vitamin C (Figure 3), but the differences were not significant.

### 3.3. DNA Strand Breaks

**Figure 7:**
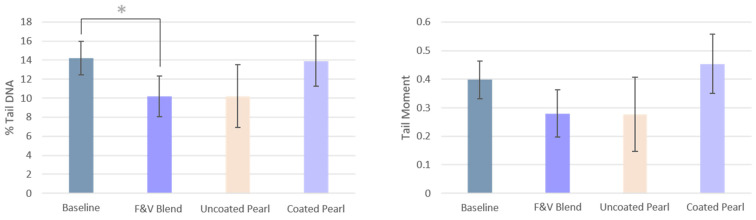
DNA damage (% Tail DNA and Tail Moment) after each DI compared to baseline. Data is presented as means ± SEM. (* = *p* < 0.05; gray asterisks indicate comparisons that remain statistically significant following LSD correction).

After consumption of the F&V Blend, DNA damage as measured by % Tail DNA significantly decreased compared to baseline (before FDR correction). No interventions led to significant changes in Tail Moment. The changes in Tail Moment were similar to those in % Tail DNA, but this measure had a larger variance (Figure 7). Surprisingly, the Uncoated Pearl group responded more similarly to the F&V Blend group, and the Coated Blend group’s results were closer to the baseline values.

Males who consumed the F&V Blend had a significantly greater reduction in % Tail DNA than females (*p* = 0.016) compared to their baselines, decreasing on average by 15.8% + 14.4% compared to females who only reduced on average by 1.3% + 11.4%.

### 3.4. Oxidative Stress Markers

#### 3.4.1. Antioxidant Capacity

**Figure 8:**
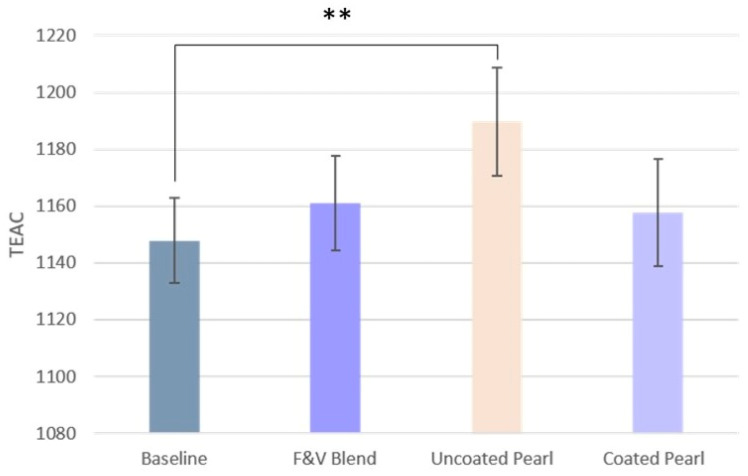
TEAC after each DI compared to baseline. Data is presented as means ± SEM. (TEAC = Trolox equivalent antioxidant capacity. ** = *p* < 0.005; statistically significant after FDR correction.

After consumption of the Uncoated Pearl, the TEAC of plasma increased significantly compared to baseline (Figure 8). Although we also see an increase in mean TEAC after consumption of the F&V Blend and the Coated Pearl, these changes were not statistically significant. The participants who consumed the interventions that contained the F&V Blend responded similarly, but in contrast to our hypothesis, those who consumed the intervention without phytochemical-rich F&Vs experienced a marked increase in TEAC.

#### 3.4.2. Superoxide Levels

There were no significant changes in the levels of superoxide anion in whole blood after the two-week consumption of any of the interventions compared to baseline (Table 4).

### 3.5. Retinal Microvasculature

**Figure 9:**
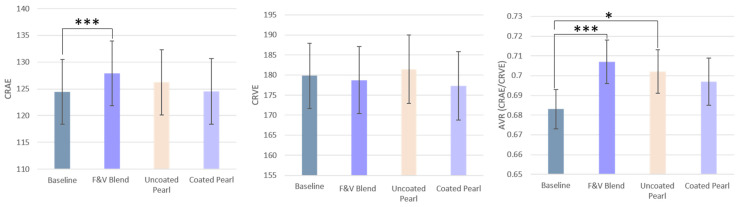
Retinal vessel caliber changes at baseline and after each intervention. Data is presented as mean ± SEM. (* = *p* < 0.05; *** = *p* < 0.001; significance following FDR correction).

Only the F&V Blend group experienced a significant increase in CRAE with an average difference of 3.51 µm (Table 4) from baseline. No significant changes in CRVE were observed between the baseline and post-intervention measurements. The AVR significantly increased for the F&V Blend group compared to baseline and for the Uncoated Pearls group (Figure 9). Overall, the biggest phenotypic improvements in retinal microvasculature were from the blended F&V product, and then the Uncoated Pearl intervention, whereas the Coated Pearl group did not differ much from baseline.

### 3.6. SNPs

The SNP frequencies within this study population were similar to the frequencies previously reported (Table 2) and the sample size per intervention group is given in Table 5.

**Figure 10:**
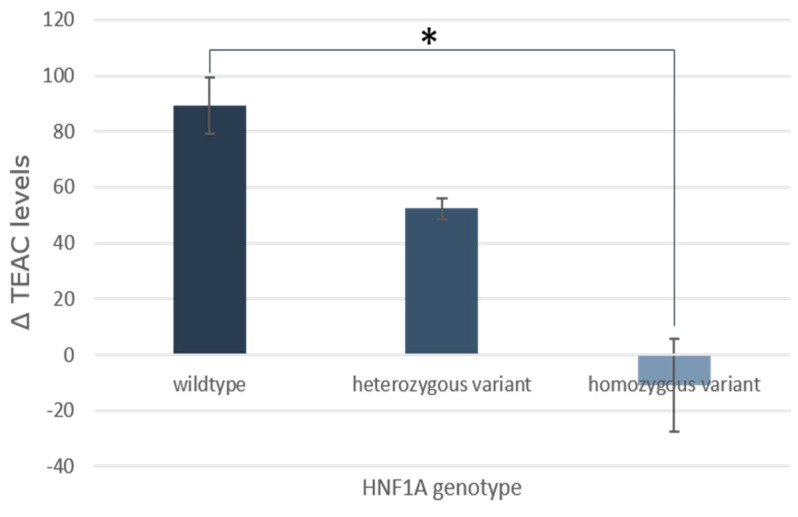
Effect of HNF1A genotype on changes in TEAC after consumption of the Uncoated Pearl compared to baseline. Data is presented as mean + SEM. (TEAC = Trolox equivalent antioxidant capacity; * = *p* < 0.05 after FDR correction).

After stratifying participants by genotype for each of the 15 SNPs and comparing the changes in % Tail DNA, Tail Moment, AVR and TEAC for each intervention group, only the Uncoated Pearl group showed significant differences for TEAC with variations in HNF1A (Figure 10). The wildtype and heterozygous subgroups showed increases in TEAC levels after the intervention, while the levels in the homozygous group decreased on average. After FDR correction, the antioxidant capacity in the wildtype group was significantly different from that of the homozygous group (*p* < 0.05).

## 4. Discussion

The primary objective of this study was to evaluate the impact of a novel fruit- and vegetable-coated dried food product on biomarkers associated with chronic disease risk, in comparison to a traditional fruit and vegetable nutrition intervention, while also accounting for genetic variation in assessing their effectiveness. After two weeks of consuming 133 g per day of the Pearls coated with 400 g of F&V, carotenoid plasma concentrations significantly increased (for lycopene, alpha-carotene, and beta-carotene), and were similar to those who consumed 400 g of the F&V per day for two weeks. However, consumption of the Coated Pearl did not result in significant improvements in other markers of disease risk. After two weeks of consuming 100 g per day of the Uncoated Pearl, composed mostly of rice starch and 5% oat flour, carotenoid and polyphenol absorption and vitamin C excretion did not increase, following expectations. Unexpectedly, the plasma antioxidant capacity and overall microvasculature diameter significantly improved in these participants. In those who consumed 400 g of the F&V Blend per day for two weeks, plasma carotenoid concentrations (lutein, alpha-carotene, and beta-carotene) significantly increased, DNA strand breaks reduced after oxidative exposure (% Tail DNA), arteriolar dilation significantly increased, and the overall retinal vessel diameter significantly improved. Additionally, when participants consumed the F&V Blend, they reported a significant increase in overall sugars and fiber intake in their food diaries, whereas those who consumed the Pearl products reported a significant increase in overall calories. The Coated Pearl group also reported more protein intake during the intervention compared to baseline, but this was only significant before FDR correction.

Several factors should be taken into account when interpreting these results. The results for the Coated Pearl group are contingent on the potential effect of the F&V Blend. The complexity of this blend, which combines a variety of F&Vs, may have diluted the dose of individual phytochemical compounds or phytochemical classes, potentially leading to less pronounced effects on disease risk biomarkers within this two-week timeframe. Simultaneous experiments with simpler F&V Blends that had a higher concentration of specific bioactive compounds have shown stronger impacts in a short duration [49]. However, given our hypothesis that the most complex blend of F&Vs and phytochemicals may induce the most pronounced impact on human physiology due to their synergistic effects, the Coated Pearls were designed with this particular F&V Blend [2,3].

Additionally, it is worth noting that the study participants were healthy individuals, so only subtle changes were expected in response to a dietary intervention. The relatively short study duration of two weeks was chosen as this is sufficient time to observe gene expression changes after a dietary intervention, but this may not have allowed adequate time to observe significant changes in certain biomarkers. Furthermore, the process of producing the Pearls appeared to increase the extractability of concentrations of various phytochemical classes, such as chlorogenic acid and catechins, while reducing others like carotenoids, leading to vitamin C loss [25]. These factors may have played a role in the absence of significant effects on certain outcomes. However, this does not explain why the Uncoated Pearl group experienced an increase in antioxidant capacity whereas the interventions that contained this F&V Blend did not. In addition, the method to measure the amounts of total polyphenols might not have been specific enough to measure differences between the intervention groups, as the Folin–Ciocalteu reagent used might interact with other substances co-extracted from plasma during the deproteinization process, such as reducing sugars, amino acids, and other proteins that may be present in the plasma. This non-specificity could contribute to an overestimation of the polyphenol content or, conversely, mask subtle differences between baseline and post-intervention measurements. Despite some limitations, the method is considered reliable and has been validated through comparisons with more specialized techniques like HPLC. Future studies with a larger sample size and perhaps a more specific, alternative method for polyphenol quantification, such as high-performance liquid chromatography (HPLC), might provide further insight into the true effects of the intervention on plasma polyphenol concentrations.

A known component of oat fiber is beta-glucans, which are polysaccharides that have a wide spectrum of biological activity. Animal studies have shown an improvement in antioxidant capacity after animals were fed oat beta-glucans [51,52]. However, the small amount of oat flour present in the intervention makes this link between the Uncoated Pearls and improved TEAC suspect. The food diary data from the participants also does not offer alternative theories, as the changes in dietary nutrients between interventions were minor and the Uncoated Pearl group does not distinctly differ aside from a slight increase in the percentage of calories from carbohydrates (2%) and slight decrease in the percentage of calories from fats (2–4%). Even the statistically significant increase in calories in the Pearl groups (Uncoated: 190 kcal/day; Coated: 214 kcal/day) is less than the total calories in the Pearls themselves (386 kcal/day). Similarly, the 16 g increase per day in sugars and 10 g increase in fiber in the F&V Blend group are partially and fully attributable to the F&V Blend (sugars: 23 g/day; fiber: 11 g/day), respectively.

The study revealed certain differences in responses that were specific to sex. Although corrected for in the overall analysis, sex was further examined for outcomes where it was a significant factor. Prior to FDR correction, the Coated Pearl group showed a significant rise in plasma lycopene levels following the intervention compared to baseline. Females in the Coated Pearl group showed an increase in plasma lycopene concentrations after the intervention (unlike the mean decrease seen in males), suggesting better absorption in females in response to this food. Males who consumed the F&V Blend had a greater improvement in resistance to ex vivo-induced DNA strand breaks (% Tail DNA) than females, who did not differ much from baseline, likely driving the mean reduction seen in this group. In contrast, although not significant, females showed a larger reduction in % Tail DNA after the Pearls than did males. Sex was also found to impact responses to oxidative DNA damage in a previous study involving a blueberry–apple juice intervention [7]. Sex-related differences in phytochemical absorption and physiological improvements from the same nutrients requires further investigation.

This study aimed to explore how genetic variation might influence changes in chronic disease risk markers following consumption of the Pearls. However, with the exception of the HNF1A gene (Figure 10), no significant differences were found between genetic sub-groups—likely due to the limited sample size. For HNF1A, individuals with the wildtype genotype experienced a notable increase in their plasma antioxidant capacity after consuming the Uncoated Pearls compared to those with the homozygous variant. HNF1A variants have previously been associated with an elevated risk of gestational and type II diabetes mellitus, primarily through their role in promoting insulin resistance [53,54]. In states of hyperinsulinemia, a precursor to insulin resistance, Nrf2 expression is suppressed via insulin’s induction of heterogeneous ribonucleoproteins F and K, leading to an impaired antioxidant and cyto-protective defense capacity [55]. Although our participants were free of metabolic disorder diagnoses, a supposed gradation in insulin concentrations in the participants according to their different HNF1A genotypes could offer an explanation for the progression in antioxidant capacity among these participants.

Some limitations of our study and future recommendations relate to our study population characteristics. In order to compare more effectively potential differences in responses among males and females, evenly matched sex groups would be ideal. However, we were still able to note some differences in the characteristics of our male and female populations and otherwise control for sex-related differences in order to provide general conclusions. Furthermore, it is essential to acknowledge the constraints imposed by studying the effects of diet interventions in a population already deemed healthy, limiting the magnitude of change that can be identified. Finally, the influence of oat fiber on antioxidant capacity, although minimal in the Pearl groups, should be taken into account when explaining unexpected findings. The F&V-coated product failed to deliver improvements beyond an increase in carotenoid plasma concentrations. This outcome could be ascribed to the limited statistical power, potential losses of critical phytochemicals during the infusion process, and/or the influence of changes in the food matrix on the products’ ability to alter markers of chronic disease risk.

## 5. Conclusions

In conclusion, this study highlights the complex nature of evaluating the effects of intricate blends of F&Vs in comparison to dry food products on markers related to chronic disease risk. The F&V Blend was more effective at improving carotenoid absorption, retinal vessel dilation, and DNA damage resistance than the processed dried food products, which were limited to improving only carotenoid absorption. The findings indicate that several factors—including the type and amount of bioactive compounds, study duration, participant group size and characteristics, and methods of food processing—are key determinants of health-related outcomes. Future studies with larger cohorts, extended follow-up periods, and optimized formulations may offer a clearer understanding of the potential advantages of these dietary strategies.

## Figures and Tables

**Table 1:** Dietary intervention compositions for F&V Blend, Uncoated Pearls and Coated Pearls, including the overrepresented phytochemicals, amount (g), and type of fruits and vegetables. Preparation described in detail in DeBenedictis et al. (2023) [25].

Dietary Intervention	Overrepresented Phytochemicals	Fruits and Vegetables
F&V Blend	Total polyphenols Anthocyanins Carotenoids Diallyl sulfide Glucosinolates	100 g apples, green tea polyphenols (0.5 g green tea in 25 mL water), 25 g blueberries, 25 g blue grape, 25 g blackberries, 25 g raspberries, 33 g tomato, 33 g carrots, 33 g sweet red pepper, 33 g broccoli, 33 g cauliflower, 33 g Brussels sprouts
Uncoated Pearls	Lacks significant concentrations of phytochemicals—mostly starch + minor beta glucan soluble fiber	95 g rice flour + 5 g oat flour
Coated Pearls	Total polyphenols Anthocyanins Carotenoids Diallyl sulfide Glycosylates	100 g apples, green tea polyphenols (0.5 g green tea in 25 mL water), 25 g blueberries, 25 g blue grape, 25 g blackberries, 25 g raspberries, 33 g tomato, 33 g carrots, 33 g sweet red pepper, 33 g broccoli, 33 g cauliflower, 33 g Brussels sprouts 95 g rice flour + 5 g oat flour

**Table 2:** Identified genetic polymorphisms to be evaluated based on their contribution to the results of dietary intervention studies on fruits and vegetables [7,34,35,36,37,38,39,40,41,42,43,44,45,46].

SNP Name	Full Name	Wildtype	Variation	Amino Acid Change	dbSNP ID	Expected Frequencies (%) ^1^			Experimental Frequencies (%) ^1^		
						Wt	Hz	Hm	Wt (n)	Hz (n)	Hm (n)
GSTM1*0	Glutathione s-transferase mu 1	Present	Deletion	Deletion	-	51.1	-	48.9	32.9 (27)	-	67.9 (55)
NQO1*2	NAD(P)H quinone dehydrogenase 1	GG	G > A/G > C	p.R139W, p.R139G	rs1800566	63	33	4	92.6 (75)	-	7.4 (6)
CAT1*1	Catalase 1	CC	C > G/C > T	N/A	rs1001179	62.7	33	4.3	63.0 (51)	33.3 (27)	3.7 (3)
GSTT1*0	Glutathione S-transferase T1	Present	Deletion	Deletion	-	73	-	27	91.4 (74)	-	8.6 (7)
XRCC1*4	X-ray repair cross-complementing protein 1	TT	T > C/T > G	p.Q399R, p.Q399P	rs25487	11.8	45.1	43	7.4 (6)	45.7 (37)	46.9 (38)
ZBED3	Zinc finger BED domain-containing protein 3	GG	G > A/G > T	N/A	rs4457053	9.2	42.3	48.5	9.9 (8)	35.8 (29)	54.3 (44)
Glu298Asp	Endothelial nitric oxide synthase	TT	T > A/T > G	p.D298E	rs1799983	8.8	41.8	49.4	4.9 (4)	40.7 (33)	54.3 (44)
COMT	Catechol-O-Methyltransferase	GG	G > A	p.V158M	rs4680	26.3	50	23.7	39.5 (32)	44.4 (36)	16.0 (13)
SLC23A1	Solute carrier family 23 member 1	TT	T > A/T > C/T > G	N/A	rs10063949	38.8	47	14.2	32.1 (26)	40.7 (33)	27.2 (22)
MTHFR	Methylenetetrahydrofolate reductase	GG	G > A/G > C	p.A263V, p.A263G	rs1801133	44	44.7	11.3	35.8 (29)	51.9 (42)	12.3 (10)
HNF1A	Hepatocyte nuclear factor-1 alpha	AA	A > C/A > T	p.I27L, p.I27P	rs1169288	45.3	44	10.7	37.0 (30)	53.1 (43)	9.9 (8)
GSTP1	Glutathione S-transferase pi 1	AA	A > G/A > T	p.I105V, p.I105P	rs1695	45	44.2	10.8	43.2 (35)	45.7 (37)	11.1 (9)
TCF7L2	Transcription factor 7-like 2	CC	C > G/C > T	N/A	rs7903146	50.8	40.9	8.2	55.6 (45)	39.5 (32)	4.9 (4)
BCMO1	Beta-carotene 15,15′-monooxygenase 1	CC	C > T	p.A379V	rs7501331	61.7	33.7	4.6	74.1 (60)	21.0 (17)	4.9 (4)
APOC1	Apolipoprotein C1	AA	A > G	N/A	rs4420638	69.1	28	2.8	77.8 (63)	21.0 (17)	1.2 (1)

^1^ Wt = wildtype; Hz = heterozygous; Hm = homozygous. Frequencies of null or deletion alleles are labeled under ‘Hm’. N/A = not available.

**Table 3:** The forward and reverse primer, and product sizes of β-globin, GSTM1*0, and GSTT1*0 for the PCR assay.

Gene	Forward Primer	Reverse Primer	Product Size (bp) *
β-globin	5′-CAACTTCATCCACGTTCACC-3′	5′-GAAGAG CCAAGGACAGGTAC-3′	268
GSTM1*0	5′-GAACTCCCTGAAAAGCTAA AGC-3′	5′-GTTGGGCTCAAATATACGGTGG-3′	215
GSTT1*0	5′-TTCCTT ACTGGTCCTCACATCTC-3′	5′-TCACCGGATCATGGCCAGCA-3′	480

* bp = base pair.

**Table 4:** Participant characteristics and outcome measures at baseline, and after a two-week intervention period where participants consumed either a fruit and vegetable blend (F&V Blend), Uncoated, or Coated Pearls.

	Baseline	F&V Blend	Uncoated Pearl	Coated Pearl
n	82	41	20	21
Age	29 ± 1	28 ± 9	32 ± 13	29 ± 11
Sex	78% female	88% female	70% female	71% female
BMI	22.8 ± 0.2	22.6 ± 1.9	22.8 ± 2.2	23.3 ± 1.9
Plasma Total Polyphenol	255 ± 3.4	255 ± 4.1	250 ± 5.1	256 ± 5.0
Plasma Lutein (nM)	271.0 ± 21.0	344.3 ± 23.3 ***	255.3 ± 28.2	288.8 ± 26.9
Plasma Lycopene (nM)	493.3 ± 29.6	537.6 ± 33.6	530.9 ± 41.62	560.7 ± 39.5 *
Plasma Alpha-carotene (nM)	117.5 ± 12.9	174.0 ± 15.8 ***	105.3 ± 21.3	209.7 ± 19.9 ***
Plasma Beta-carotene (nM)	637.4 ± 68.1	912.4 ± 73.1 ***	652.5 ± 84.0	898.4 ± 81.1 ***
Urinary Vitamin C (mg/mL)	0.77 ± 0.07	0.79 ± 0.09	0.73 ± 0.11	0.72 ± 0.11
% Tail DNA	14.23 ± 1.76	10.17 ± 2.14 *	10.20 ± 3.3	13.91 ± 2.67
Tail Moment	0.40 ± 0.066	0.28 ± 0.082	0.28 ± 0.13	0.45 ± 0.10
TEAC levels	1148 ± 2	1161 ± 3	1190 ± 4 **	1158 ± 4
Superoxide anion height	3259 ± 27	3735 ± 45	2858 ± 83	3186 ± 80
Superoxide anion area	1,217,296 ± 10,095	1,216,606 ± 16,626	1,019,482 ± 28,842	1,167,825 ± 27,612
CRAE	119.96 ± 4.61	125.05 ± 4.66 ***	122.74 ± 4.75	121.34 ± 4.74
CRVE	172.17 ± 5.45	174.73 ± 5.68	175.88 ± 6.04	173.37 ± 6.0
AVR	0.68 ± 0.01	0.71 ± 0.1 ***	0.70 ± 0.01 *	0.70 ± 0.01
Calories (kcal)	1755 ± 58	1835 ± 64	1945 ± 76 *	1969 ± 77 *
Carbohydrates (g) [% of kcal]	211 ± 12 [48%]	220 ± 14 [48%]	243 ± 18 [50%]	238 ± 18 [48%]
Proteins (g) [% of kcal]	83 ± 4 [19%]	85 ± 4 [19%]	84 ± 5 [17%]	96 ± 5 * [19%]
Fats (g) [% of kcal]	68 ± 4 [35%]	66 ± 4 [32%]	60 ± 5 [28%]	66 ± 5 [30%]
Cholesterol (mg)	139 ± 24	151 ± 27	162 ± 31	146 ± 32
Sodium (mg)	842 ± 117	889 ± 129	789 ± 150	667 ± 152
Sugars (g)	43 ± 4	59 ± 4 ***	47 ± 5	44 ± 5
Fibers (g)	12 ± 1	22 ± 1 ***	11 ± 1	13 ± 1

Data is presented as means ± SEM. Significant differences indicated for comparisons between baseline and post-intervention tests. * = *p* < 0.05, ** = *p* < 0.005, *** = *p* < 0.001. Total asterisks represent *p*-values before FDR correction, and black asterisks represent *p*-values after FDR correction.

**Table 5:** Experimental frequency of participant genotypes by intervention group.

SNP Name	F&V Blend			Uncoated Pearl			Coated Pearl		
	Wt (n)	Hz (n)	Hm (n)	Wt (n)	Hz (n)	Hm (n)	Wt (n)	Hz (n)	Hm (n)
GSTM1*0	13	-	28	9	-	11	5	-	16
NQO1*2	40	-	1	17		3	18		2
CAT1*1	25	15	1	12	8	-	14	4	2
GSTT1*0	39	-	2	18	-	2	17	-	3
XRCC1*4	5	18	18	-	11	9	1	8	11
ZBED3	4	14	23	2	8	10	2	7	11
Glu298Asp	2	19	20	-	6	14	2	8	10
COMT	18	17	6	6	11	3	8	8	4
SLC23A1	15	21	5	8	5	7	3	7	10
MTHFR	19	17	5	4	13	3	6	12	2
HNF1A	17	23	1	5	12	3	8	8	4
GSTP1	19	18	4	6	11	3	10	8	2
TCF7L2	25	14	2	8	11	1	12	7	1
BCMO1	32	8	1	15	3	2	13	6	1
APOC1	34	6	1	14	6	-	15	5	-

Wt = wildtype, Hz = heterozygous, Hm = homozygous.

## Data Availability

The raw data supporting the conclusions of this article will be made available by the authors on request.

## Abbreviations

The following abbreviations are used in this manuscript:

F&V	fruits and vegetables
SNP	single nucleotide polymorphism
TEAC	Trolox equivalent antioxidant capacity
ICTRP	International Trial Registry Platform
ESR	electron spin resonance spectroscopy.
CRAE	central retinal artery equivalent
CRVE	central retinal vein equivalent
AVR	arteriolar-to-venular ratio
DI	dietary intervention
MUMC+	Maastricht University Medical Center +
Bp	base pair
Wt	wildtype
Hz	heterozygous
Hm	homozygous
